# Association of a Common Oxytocin Receptor Gene Polymorphism with Self-Reported ‘Empathic Concern’ in a Large Population of Healthy Volunteers

**DOI:** 10.1371/journal.pone.0160059

**Published:** 2016-07-28

**Authors:** Franz Korbinian Huetter, Hagen Sjard Bachmann, Anette Reinders, Doris Siffert, Patrick Stelmach, Dietmar Knop, Peter Alexander Horn, Winfried Siffert

**Affiliations:** 1 Institut für Pharmakogenetik, Universitätsklinikum Essen, Universität Duisburg Essen, D-45122 Essen, Germany; 2 Institut für Transfusionsmedizin, Universitätsklinikum Essen, Universität Duisburg Essen, D-45122 Essen, Germany; French National Centre for Scientific Research, FRANCE

## Abstract

**Background:**

Previous research has linked genomic variations of the oxytocin receptor (*OXTR*) gene with individual differences in empathy. The impact of these variations on specific cognitive and emotional aspects of empathy, however, remains to be clarified.

**Methods:**

We analysed associations of a common *OXTR* polymorphism (rs53576) with trait empathy in a sample of 421 blood donors (231 M, 190 F; age 18–74) using the Interpersonal Reactivity Index (IRI) as an established multidimensional self-report measure of empathy.

**Results:**

Female sex was significantly associated with higher empathy scores in all IRI scales (p<0.001) with the exception of the cognitive perspective taking scale (p = 0.09). The overall trait empathy score was significantly associated with rs53576 (p = 0.01), with mean scores increasing from AA to GG genotypes. An analysis of the IRI subscores revealed that the polymorphism was especially associated with the emotional empathic concern scale (p = 0.02). Separate analysis of the male and female subgroup revealed a significant association of the polymorphism with female (p = 0.04), but not with male (p = 0.20) empathic concern. A comparison of effect sizes between the groups showed greater effects for women compared to men although effect size differences did not become significant in our sample.

**Conclusions:**

Our findings suggest a significant association of the rs53576 *OXTR* gene polymorphism with trait empathy and especially with emotional aspects of empathy. This association is possibly weaker or absent in men compared to women.

## Introduction

Empathic skills and the regulation of one’s empathic responses to fellow human beings are widely recognized to be an important prerequisite of social and psychological functioning. Empathic reactions to another person’s experience involve a cognitive dimension, such as seeing a situation from the other person’s perspective, and an affective dimension, enabling an individual to share to some extent the emotions of another and to feel motivated for supportive action [1]. Specific empathic deficits have been identified in a variety of psychiatric conditions including psychopathy [2], attention-deficit/hyperactivity disorder [3], schizophrenia [4] and autism spectrum disorders [5]. Such clinically relevant empathic deficits have placed the question of physiological correlates and biomarkers of empathic processes into the focus of research. Due to the abundant evidence linking the oxytocin system to attachment and social affiliation in humans [6], genetic variations of the oxytocin receptor (*OXTR*) gene have been a prominent subject of studies on the biological determinants of empathy [7, 8]. Several polymorphisms of the *OXTR* gene have been shown to modify the function of the oxytocin system in terms of personality and social behaviour [9]. Among these, the rs53576 polymorphism, a silent G to A substitution in the third intronic region, is one of the most intensively studied variations. A meta-analysis of 24 studies (n = 4955) [10] found that the GG genotype is associated with more general sociality, including traits such as extraversion, trust and empathy, compared to the GA and AA genotypes. The impact of these variants on specific cognitive and emotional aspects of empathy, however, remains to be clarified.

In this study, we, therefore, set out to investigate associations between the rs53576 polymorphism and dispositional empathy using Davis’ Interpersonal Reactivity Index (IRI) [1, 11], a multidimensional self-report questionnaire that covers both cognitive and emotional aspects of empathy, including empathic responses with a more other-oriented (“empathic concern”) and a more self-oriented (“personal distress”) tendency.

The results of the few studies associating IRI scores with rs53576 genotypes so far are inconsistent. Wu, Li & Su [9] did not find associations for this polymorphism in a sample of 101 non-clinical Chinese subjects nor did Montag et al. [12] in a sample of 145 German schizophrenic patients and 145 healthy controls. On the other hand, Smith et al. [13] found a significant association between the rs53576 GG genotype and the empathic concern scale of the IRI in a sample of 51 male participants between 18 and 35 years of age. Likewise, in a study with 367 young adults, Uzefovsky et al. [14] found that the rs53576 polymorphism was associated with a cumulative emotional empathy score calculated from three empathy tests including the IRI.

We designed our study to comprise a large, heterogeneous sample of mixed age and gender. Considering the sex differences in empathy and in the effects of *OXTR* gene variants found in previous research [15], this also allowed us to analyse associations between genotype and empathy for men and women separately with sufficient group sizes.

## Materials and Methods

### Ethics statement

The study was approved by the local ethics committee (University of Duisburg-Essen, Medical Faculty, reference number 14-5797-BO). All participants gave written informed consent prior to testing. Data were acquired anonymously, with age and sex being the only demographic data collected in the header section of the questionnaire.

### Participants, data and sample acquisition

500 participants were recruited from a population of blood donors at the Institute for Transfusion Medicine of the Essen University Hospital from January to April 2015. Participants were asked to fill in the IRI test prior to blood donation (for the German questionnaire, please, refer to S1 File). Upon completion, participants handed back the questionnaire and were given an EDTA tube which was matched with the questionnaire by a number code. Blood samples were taken by the blood bank staff and handed back to us in bulk to grant for anonymity.

IRI test results of participants from whom no blood samples could be acquired, for example, because they failed to meet the requirements to donate blood–were excluded from the evaluation. Equally, questionnaires with incomplete or ambiguous answers were excluded. As a result, 421 questionnaires and matched blood samples were evaluated (for the raw data, please, refer to S2 File).

The 421 participants (231 male / 190 female) were between 18 and 74 years of age, with a mean age of 36.6 years and a median age of 30 years. Male participants’ age-span ranged from 18 to 74 years, with a mean age of 38.9 and a median age of 35 years. Female participants were between 18 and 73 years of age, with a mean age of 33.7 and a median age of 25 years.

### Measure of empathy

We used the German version of Davis’ 28-item Interpersonal Reactivity Index (IRI) [16]. The IRI consists of four subscales: The main cognitive subscale is **perspective taking (PT),** describing a disposition to adopt other people’s point of view. The **fantasy (FT)** scale refers to an inclination to identify with characters in movies, novels, and other fictional genres. It was originally considered to be an instance of emotional empathy by Davis [1], but is attributed to the cognitive dimension in more recent research [9,17]. Clearly emotional dimensions of the IRI are empathic concern (EC) and personal distress (PD). The **empathic concern (EC)** scale assesses the degree of other-oriented emotional empathy, especially a tendency towards compassionate care and feelings of warmth for other individuals experiencing negative situations. High EC scores have been shown to be strongly associated with prosocial behaviour [18] and with helping others even at the expense of own disadvantages [19]. In contrast, **personal distress (PD)** refers to a more self-oriented reaction to others in distress, including anxiety, helplessness and a tendency to avoid the discomforting situation [1].

### Determination of the *OXTR* rs53576 genotypes

Isolation of genomic DNA from blood was performed using a commercially available kit (QIAamp DNA Blood Mini Kit, Qiagen, Hilden, Germany) following the manufacturer’s instructions. Genotypes of the rs53576 polymorphism were determined by PCR using the following primers: forward primer, 5`-CCACATCACTGGGTCACCTC-3`and reverse primer, 5`-AGGAGGCCTGGTTTGAACTG-3`. After an initial denaturation at 95°C, 35 cycles of DNA amplification were done using Ampliqon Mastermix (Odense M, Denmark) at 95°C for 30s, 63°C for 30s, 72°C for 30s 72°C for 10 min. The 185-bp PCR products were digested using the restriction enzyme *Bam*HI Fast Digest (Thermo Fisher Scientific, Waltham, MA USA) and analysed on a 3% agarose gel. The unrestricted products (185-bp) represented the GG genotype, the completely restricted products (106 bp + 79 bp) represented the AA genotype.

### Statistics

In line with current research [9,20,21], we calculated cumulative scores from the IRI subscales. We calculated a trait empathy score (TES) as the sum of all individual IRI scores (TES = PT+FT+EC+PD), a cognitive empathy score (CES) as the sum of perspective taking and fantasy (CES = PT+FT) and an emotional empathy score (EES) as the sum of empathic concern and personal distress (EES = EC+PD). Descriptive measures of location and variation were calculated for the cumulative scores and for the four IRI subscales. In addition to tests for skewness and kurtosis, a Kolmogorov-Smirnov test with Lillefors significance correction was performed to test for normal distribution. As the normality-assumption was not met for most of the scores, non-parametric testing was applied to test for associations of IRI scores with genotype and gender. Based on previous evidence on the linear effect of AA to GA to GG genotypes on levels of empathy [10], we hypothesized an allele-dosage-effect and interpreted the genotypes as ordinal rather than nominal variables. As the Jonckheere-Terpstra-Test, which tests for medians ordered by magnitude, has previously been shown to be more precise for genotype-effects with an *a priori* order of alternatives compared to the commonly used Kruskal-Wallace-Test [22], we used the Jonckheere-Terpstra-Test to test for associations between IRI scores and genotypes. Mann-Whitney-U-Test was used to analyse the impact of sex on empathy scores. Contingency tables and Pearson’s Chi-squared tests were used to test for possible gene-by-sex and gene-by-age interactions. Compliant with our non-parametric approach, we calculated effect sizes on the basis of Spearman's rank correlation coefficients to express the effects of genotype on empathy for the complete sample and by sex. Significance of between-group correlation differences was calculated using a public domain software [23] based on Fisher [24]. Power and sample size analyses were performed using Monte Carlo simulations based on Kendall and Gibbons [25] and Devroye [26]. Control for deviation from the Hardy-Weinberg equilibrium was conducted using a public domain software. Allele frequency was compared to a sample of 503 subjects of European descent using data available at www.1000genomes.org [27].

Differences were regarded significant at p<0.05. All statistical analyses were done using SPSS 23.0.

## Results

### Genotype distribution

The distribution of the rs53576 genotypes were in accordance with the Hardy-Weinberg equilibrium for the complete sample as well as for males and females alone. The A-allele frequency of 34.1% was comparable to a sample of 503 subjects of European descent with a frequency of 35.1% [27]. Chi-squared tests revealed no significant associations between allele frequencies and sex as well as age.

### Genotype and trait empathy

Results for the trait empathy score (TES) are given in **Table 1.** The TES was significantly associated with genotype (p = 0.01), with mean scores increasing from AA to GG genotypes (mean TES score ± SD: AA 58.1 ± 9.3, GA 58.9 ± 10.5, GG 61.1 ± 10.4). Spearman's correlation between genotype and TES was r = .120, p = 0.01, 95% CI [0.02, 0.21], r^2^ = 0.014, accounting for 1.4% of the variability in TES ranks.

**Table 1:** Trait empathy score by rs53576 genotype for all participants.

	All			AA			GA			GG			p*
	(n = 421 | 100%)			(n = 54 | 12.8%)			(n = 179 | 42.5%)			(n = 188 | 44.7%)			
	Mean	SD	Median	Mean	SD	Median	Mean	SD	Median	Mean	SD	Median	
**TES**	59.8	10.4	60	58.1	9.3	57	58.9	10.5	59	61.1	10.4	61	0.01

TES: trait empathy score calculated as the sum of perspective taking (PT), fantasy (FT), empathic concern (EC) and personal distress (PD) scores.

* The *p* value were calculated using the Jonckheere-Terpstra-Test.

### Genotype and cognitive vs. emotional empathy

A more fine-grained analysis distinguishing between cognitive and emotional aspects of empathy showed an increase both for the cognitive empathy score (mean CES score ± SD: AA 31.0 ± 6.3, GA 31.2 ± 6.8, GG 32.5 ± 6.7; p = 0.10) and the emotional empathy score (mean EES score ± SD: AA 27.2 ± 4.9, GA 27.6, ± 5.9; GG 28.6, ±5.9; p = 0.04). The association between genotype and empathy score, however, was only significant for emotional empathy as shown in **Table 2.** Spearman's correlation between genotype and EES was r = .104, p = 0.03, 95% CI [0.01, 0.02], r^2^ = 0.014, accounting for 1,4% of variability in the EES ranks, while the correlation between genotype and CES was not significant: r = .081, p = 0.10, 95% CI [-0.01, 0.18], r^2^ = .007.

**Table 2:** Cognitive and emotional empathy scores by rs53576 genotype for all participants.

	All			AA			GA			GG			p*
	(n = 421 | 100%)			(n = 54 | 12.8%)			(n = 179 | 42.5%)			(n = 188 | 44.7%)			
	Mean	SD	Median	Mean	SD	Median	Mean	SD	Median	Mean	SD	Median	
**CES**	31.8	6.7	32	31.0	6.3	31	31.2	6.8	32	32.5	6.7	32	0.10
**EES**	28.0	5.8	28	27.2	4.9	27	27.6	5.9	28	28.6	5.9	29	0.04

CES: cognitive empathy score calculated as the sum of perspective taking (PT) and fantasy (FT) scores. EES: emotional empathy score, calculated as the sum of empathic concern (EC) and personal distress (PD) scores.

* *p* values were calculated using the Jonckheere-Terpstra-Test.

### Genotype and IRI subscores

An analysis of the IRI subscores is given in **Table 3.** As for the cumulative CES score, none of the two subscores of cognitive empathy (PT: perspective taking and FT: fantasy) was significantly associated with rs53576 genotype. Of the emotional dimensions (EC: empathic concern and PD: personal distress), only EC was significantly associated with genotype (p = 0.02), while the PD dimension was far from significant (p = 0.59). Spearman's correlation between genotype and EC was r = .111, p = 0.02, 95% CI [0.02, 0.20], r^2^ = 0.012, accounting for 1,2% of variability in the EC ranks. All other correlations were not significant.

**Table 3:** IRI subscores by rs53576 genotype for all participants.

	All			AA			GA			GG			p*
	(n = 421 | 100%)			(n = 54 | 12.8%)			(n = 179 | 42.5%)			(n = 188 | 44.7%)			
	Mean	SD	Median	Mean	SD	Median	Mean	SD	Median	Mean	SD	Median	
**PT**	17.3	3.8	18	17.2	3.7	18	17.2	3.9	17	17.5	3.8	18	0.44
**FT**	14.5	4.7	14	13.8	5.0	13	14.1	4.8	14	15.0	4.5	14	0.06
**EC**	17.0	3.0	17	16.5	2.7	17	16.7	3.0	17	17.4	3.1	17	0.02
**PD**	11.1	4.2	11	10.7	3.6	11	10.9	4.4	11	11.3	4.1	12	0.29

PT: perspective taking, FT: fantasy, EC: empathic concern, PD: personal distress

* *p* values were calculated using the Jonckheere-Terpstra-Test.

### Sex and empathy scores

As expected, the sex differences concerning empathy scores reported in previous research [1] were replicated in our sample (see **Table 4**). Female participants scored higher in all IRI dimensions than male participants. These differences did not reach a level of significance for perspective taking (PT: p = 0.09), but for all other IRI dimensions (p < 0.001). Spearman's correlations of empathy scores and gender were significant (p = 0.001) for all scores but for perspective taking (p = 0.09). Significant effects ranged from r = .292 for EC and r = .439 for TES, accounting for between 9 to 19% of variability in empathy ranks.

**Table 4:** IRI test results for all participants by sex.

	All			Male			Female			p*
	(n = 421)			(n = 231)			(n = 190)			
	Mean	SD	Median	Mean	SD	Median	Mean	SD	Median	
**TES**	59.8	10.4	60	55.8	9.4	56	64.7	9.3	65	< 0.001
**CES**	31.8	6.7	32	29.9	6.5	30	34.0	6.4	34	< 0.001
**PT**	17.3	3.8	18	17.0	4.0	17	17.7	3.6	18	0.09
**FT**	14.5	4.7	14	12.9	4.44	13	16.3	4.4	16	< 0.001
**EES**	28.0	5.8	28	25.8	5.3	26	30.7	5.2	31	< 0.001
**EC**	17.0	3.0	17	16.1	2.99	16	18.0	2.8	18	< 0.001
**PD**	11.1	4.2	11	9.7	3.9	9	12.8	3.9	13	< 0.001

TES: trait empathy score, CES: cognitive empathy score, PT: perspective taking; FT: fantasy; EES: emotional empathy score. EC: empathic concern; PD: personal distress.

* *p* values were calculated using the Mann-Whitney-U-Test.

### Association of genotype and empathy by sex

**Fig 1:**
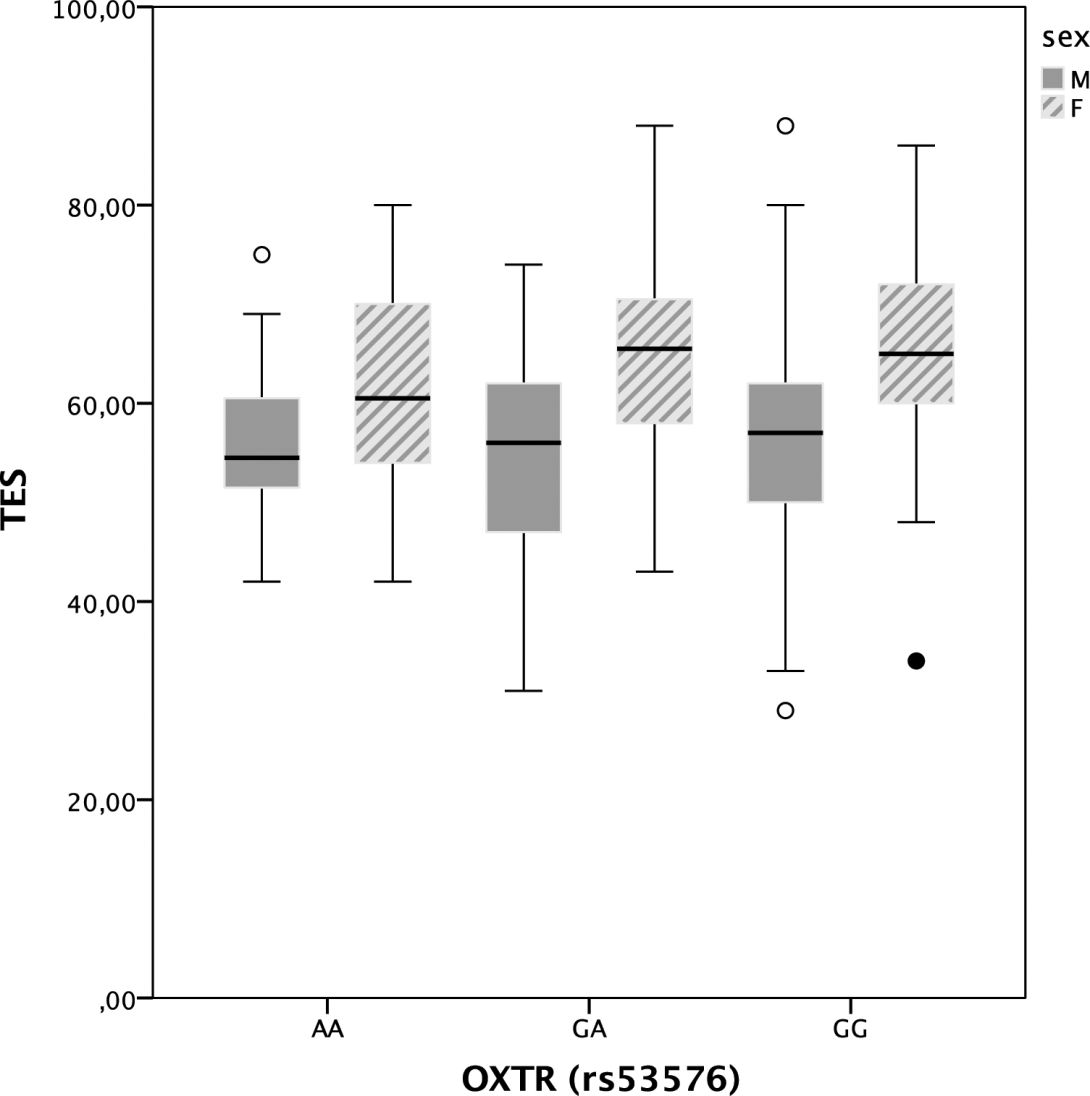
Trait empathy score (TES) across genotypes by sex. Male sex is indicated by dark grey solid boxes, female sex by light grey patterned boxes. Boxes represent the 25th percentile, the median and the 75th percentile. Whiskers represent the minima and maxima of TES scores excluding outliers.

As shown in **Fig 1,** a first analysis of the association of genotype and empathy by sex suggested a genotype by sex interaction.

Due to the possible sex differences, we analysed male and female subgroups separately to test for a possible sexually dimorphic impact of genotype on empathy. Of note, none of the IRI scores was significantly associated with rs53576 genotype for men alone (see **Table 5**).

**Table 5:** Empathy scores by rs53576 genotype for male participants.

	All			AA			GA			GG			p*
	(n = 231 | 100%)			(n = 28 | 12.1%)			(n = 103 | 44.6%)			(n = 100 | 43.3%)			
	Mean	SD	Median	Mean	SD	Median	Mean	SD	Median	Mean	SD	Median	
**TES**	55.7	9.4	56	55.2	7.7	54.5	54.7	9.5	56	57.0	9.8	57	0.15
**CES**	29.9	6.5	30	29.6	5.6	29.5	29.2	6.6	30	30.8	6.6	30	0.37
**PT**	17.0	4.0	17	17.3	3.8	18	16.7	4.1	17	17.3	3.9	17	0.73
**FT**	12.9	4.4	13	12.3	4.6	11.5	12.5	4.7	12	13.5	4.1	13	0.10
**EES**	25.8	5.3	26	25.6	3.6	25	25.4	5.4	26	26.2	5.5	26	0.25
**EC**	16.1	3.0	16	15.8	2.8	16	16.0	3.0	16	16.4	3.1	16	0.20
**PD**	9.7	3.9	9	9.8	3.0	10	9.5	4.1	9	9.8	3.9	10	0.73

TES: trait empathy score, CES: cognitive empathy score, PT: perspective taking; FT: fantasy; EES: emotional empathy score. EC: empathic concern; PD: personal distress. Medians were included to show ranked effects.

* *p* values were calculated using the Jonckheere-Terpstra-Test.

In the female subgroup (see **Table 6**), only empathic concern (EC) showed a significant association with genotype (p = 0.04). With an effect size of r = .146, p = 0.04, 95% CI [0.004, 0.283], it accounted for 2.13% of the variability in female empathic concern ranks.

**Table 6:** Empathy scores by rs53576 genotype for female participants.

	All			AA			GA			GG			p*
	(n = 190 | 100%)			(n = 26 | 13.7%)			(n = 76 | 40.0%)			(n = 88 | 46.3%)			
	Mean	SD	Median	Mean	SD	Median	Mean	SD	Median	Mean	SD	Median	
**TES**	66.7	9.3	65	61.3	9.9	60.5	64.5	9.1	65.5	65.8	9.3	65	0.06
**CES**	34.0	6.4	34	32.5	6.8	33	33.9	6.3	34	34.5	6.4	35	0.19
**PT**	17.7	3.6	18	17.0	3.7	16.5	17.8	3.6	18	17.8	3.7	18	0.44
**FT**	16.3	4.4	16	15.4	5.1	14.5	16.2	4.3	17	16.7	4.2	16.5	0.30
**EES**	30.7	5.2	31	28.8	5.5	29	30.6	5.1	31	31.3	5.0	31	0.11
**EC**	18.0	2.8	18	17.3	2.4	17	17.6	2.8	18	18.4	2.8	18	0.04
**PD**	12.8	3.9	13	11.5	3.9	11.5	13.0	4.0	13	12.9	3.6	13	0.44

TES: trait empathy score, CES: cognitive empathy score, PT: perspective taking; FT: fantasy; EES: emotional empathy score. EC: empathic concern; PD: personal distress. Medians were included to show ranked effects.

* *p* values were calculated using the Jonckheere-Terpstra-Test.

A test for the significance of difference [23, 24] of genotype effects on empathy between males and females did not, however, show a significant difference. We calculated a required sample size [25, 26] of 554 women and 932 men to achieve 80% power in contrasting the genotype effects between males and females.

## Discussion

### Association of empathic concern with rs53576 genotype

Our study shows an association of the rs53576 *OXTR* polymorphism with overall empathy expressed by a cumulative Trait Empathy Score. As previous research had shown that some *OXTR* polymorphisms have a stronger association with cognitive compared or emotional aspects of empathy [9], we contrasted the cumulative score for the two cognitive IRI dimensions (perspective taking and fantasy) with the cumulative score for the two emotional IRI dimensions (empathic concern and personal distress) and found that effects were greater and only significant for the emotional score. Further analyses showed that of the two emotional dimensions only empathic concern showed a significant effect. This differential association is plausible considering the fact that empathic concern and personal distress represent two considerably different emotional and behavioural phenotypes of emotional empathy, namely, a disposition to feel and act compassionately with other people in distress and a tendency to avoid the discomfort caused by such situations. An analysis of the male and female subgroups showed a significant association of genotype with this trait in female (p = 0.04), but not in male participants (p = 0.20). Although these findings might be indicative of a sexually dimorphic effect, it is important to note three issues:

1. Firstly, our findings do not exclude a possibly smaller effect of the polymorphism on men. In fact, our data showed an absolute increase of men’s mean EC scores from AA to GA (0.2) and from GA to GG (0.4) genotypes. But the association was not significant and the increase was smaller than in the complete sample (AA to GA: 0.2 and GA to GG: 0.7, p = 0.02) which was in turn smaller than in the female group (AA to GA: 0.3 and GA to GG 0.8, p = 0.04). It is, therefore, possible that the sexual dimorphism consists of a stronger impact of the *OXTR* variation on women compared to men rather than in having an exclusive effect on women only. Nevertheless, our findings are in contrast with those reported by Smith et al. [13], who found a significant association of the rs53576 polymorphism with empathic concern (EC) in a single-sex sample of 51 men (GG: *M* = 20.96, *SD* = 4.894; AA/GA: M = 17.89, sd = 3.935; Cohen's d = 0.69, effect size r = .33). Considering the fact that we had more than four times the number of male participants compared to Smith et al., and that there were more men (n = 231, 54.9%) than women (n = 190, 45.1%) in our sample, our results cannot be attributed to a smaller male sample size or an underrepresentation of men. Given the effect size reported by Smith et al., our sample of 231 men achieved >95% power to detect this effect (calculation based on [25, 26].
2. Secondly, the significance of differences of genotype effect sizes between men and women could not be established in this paper.
3. Thirdly, due to emerging evidence about the influence of cultural and ethnic variables on the effects of the rs53576 polymorphism on personality and behaviour [28], generalisation of such results must be considered with caution and requires testing in different cultures and ethnicities.

We are aware of the fact that the reported effects are small, which was expectable for two reasons:

1. Previous research suggests a polygenetic determination of dispositional empathy. Other polymorphisms of the oxytocin receptor such as rs237887, rs4686302, rs2268491, rs2254298 [9] as well as variations in AVPR1a [14], CD38 [29], 5-HTTPLR [30], MAO-A [31], DRD4 [32], COMT [29] genes have been shown to have an impact on empathy. This impact is often specific to certain aspects of empathy (such as emotional aspects previously associated with rs237887 and rs4686302 polymorphisms and cognitive aspects associated with rs2268491 and rs2254298 polymorphisms) and is subject to different degrees of genotype by gender interaction [9].
2. Moreover, the gene-environment interaction proves to be a decisive factor influencing the formation of empathic traits [33].

In the face of a polyetiological situation like this, it is plausible, that a single variation would have a limited effect on a complex disposition such as empathy. Further research is needed to develop an understanding of gene by gene, gene by sex and gene by environment interactions which are likely to contribute to the formation of empathic capacities.

### Strengths and limitations of our sample

Although our sample of 421 blood donors represents a specific portion of the population, it is, to the best of our knowledge, the biggest set of data so far used to investigate associations between IRI empathy scores and the rs53576 *OXTR* polymorphism. As age has been shown to have an influence on empathy across the adult life-span [34], the wide age-span of 56 years (from 18 to 74 years of age) can be considered to be a strength in terms of life-time representativeness compared to the common samples of college students. However, it may be questioned, whether blood donors are representative of the general population in terms of empathy. It is rather likely that a part of the motivation for donating blood arises from an inclination to put oneself in the position of persons in need for a blood transfusion and an altruistic intention to help them, which could result in higher scores for perspective taking (PT) and empathic concern (EC). Such an empathic bias, however, was not observed in our data. In fact, participants did not score higher in any of the four IRI dimensions than the participants in Davis’ [1,11] normative samples. This may be due to the fact, that the blood donors receive remuneration for their donation, so that the altruistic motive might, at least partly, be counterbalanced by a monetary one. A further bias might be introduced by the fact that persons with specific life style choices are excluded form donating blood in Germany according to the guidelines of the German Medical Association [35] Among them are all persons with a putative higher risk of hepatitis or HIV infections, for example persons with frequently changing sexual partners, men who had sexual intercourse with other men and alcohol or drug addicts.

### Strengths of the IRI as a measure of empathy

Although the results of the present study should also be replicated using other measures of empathy, the multi-dimensional approach of the IRI has considerable advantages for a fine-grained analysis of highly selective genotype effects on cognitive and emotional empathy. The IRI is a well-established empathy test with a satisfactory internal reliability (α-coefficients between .68 and .79), test-retest reliability (correlations between .61 and .81) and convergent validity established by its correlations with other empathy tests [1,11]. Notably, sex differences were explicitly considered in the test-design and questions were selected to load equally heavily on both sexes [11]. Moreover, self-reported empathy in the IRI has been shown to be a good predictor, not only of self-concepts, but of actual empathic performance, measured in the Reading the Mind in the Eyes test (RMET) and other empathy performance tests. Rodrigues et al. [36], for example, found that individuals homozygous for the G allele were 22.7% less likely to make a mistake in the RMET than A allele carriers and, at the same time, reported higher levels of dispositional empathy in the IRI.

### Compatibility of our results with previous research

The fact that women score higher in all IRI dimensions than men is in line with a substantial body of evidence in previous research, including the samples reported by Davis [1,11] and numerous other IRI-based studies of various age-groups and cultural backgrounds (see e.g. [37]). Of note, in our sample, the differences in perspective taking (PT) are only marginal and do not reach a level of significance (p = 0.09), which is also consistent with the results found by Davis [1,11]. These results seem to corroborate psychological research dating back to the 1970s [38] which had established in 16 of 16 samples that sex differences in empathy rather pertain to the affective reaction to other people’s experience than to the cognitive understanding of their perspective.

The specific impact of the rs53576 polymorphism on empathic concern scores is also consistent with previous research. Apart from the above mentioned study by Smith et al. [13], Uzefovsky et al. [14] recently found this trait-specificity. In this study, 367 young adults were also genotyped for the rs53576 polymorphism. In addition, they were screened with three empathy tests including the IRI, from which cumulative cognitive and emotional empathy scores were calculated. The authors found that the rs53576 polymorphism was only associated with emotional empathy, with the A allele predicting lower emotional, but not lower cognitive empathy.

The sex-specific impact of the rs53576 polymorphism on empathic concern is supported by a growing body of evidence investigating sexually dimorphic effects of *OXTR* variants. Stankova et al. [39] reported such sex-specific effects of the rs237900 and rs237902 *OXTR* polymorphisms on harm avoidance. Sexually dimorphic effects of the rs53576 polymorphism were found by Moons et al. [40], who detected an impact of rs53576 genotypes on positive affect and post-stressor salivary oxytocin (OT) levels in a Trier Stress Test paradigm in female, but not in male participants. In a study of 1000 twin children and their parents from the Michigan State University Twin Registry, Klahr et al. [41] found that the rs53576-G genotype of mothers, but not of fathers was strongly associated with parental warmth observed in s standardised videotaped interaction paradigm, even after correction for the child’s genotype.

A recently proposed endophenotype approach [42] postulating neuroplastic effects of genotypes in key oxytonergic structures of the brain is supported by fMRI-based genetic imaging studies. These studies reported significant genotype-by-sex interactions for the rs53576 polymorphism with effects on hypothalamus and amygdala grey matter volumes and resting state functional connectivity, corresponding with psychometrically measured scores in prosocial temperament [43,44]. Such findings have also been linked with the differential effect of gonadal steroids on the plasticity of oxytocinergic brain structures [15]. Although this line of research is still in its infancy and some conclusions drawn from it are still putative, its results add plausibility to the fact that sex-differences need to be taken into account in the analysis of genotype effects of the oxytocin system on empathy and other socioemotional capacities in future research.

## Conclusions

The present study has identified a significant association of rs53576 OXTR genotype with dispositional empathy in the so far largest IRI-based association study linking this polymorphism with trait empathy. A separate analysis of male and female subgroups showed a significant association for female, but not for male participants. These results may suggest a sexually dimorphic effect of the polymorphism on empathy. This hypothesis is compatible with recent research identifying a sexually dimorphic influence of the rs53576 polymorphism on the structure of key oxytonergic regions in the brain. Nevertheless, even greater sample sizes would be needed to test for significant differences in genotype effects between men and women. Our findings extend previous research on the differential effects of *OXTR* polymorphisms on empathy and invite future studies to consider sex differences in their study designs.

## Supporting Information

S1 File Questionnaire. German IRI questionnaire used in this study.
(PDF)

S2 File Study data. Spreadsheet containing all individual data, calculated IRI scores and a legend.
(XLSX)
