# Supplementary material for: Association of a Common Oxytocin Receptor Gene Polymorphism with Self-Reported ‘Empathic Concern’ in a Large Population of Healthy Volunteers
Source: PLoS One. 2016 Jul 28;11(7):e0160059. doi: 10.1371/journal.pone.0160059 (PMC4965009; doi:10.1371/journal.pone.0160059)
Supplement: S1 File — German IRI questionnaire used in this study. (PDF) [file pone.0160059.s001.PDF]

Universitätsklinikum Essen	Empathietest Auswertung	Institut für Pharmakogenetik

Lfd. Nr.: EMP - 0011 /w/48	nie	selten	manchmal	oft	immer	Ergebnis
1. Ich habe ziemlich regelmäßig Tagträume und Fantasien über Dinge die mir passieren könnten. FT	0	1	2	3	4	
2. Ich empfinde warmherzige Gefühle für Leute, denen es weniger gut geht als mir. EE	0	1	2	3	4	
3. Manchmal fällt es mir schwer, die Dinge vom Standpunkt des anderen zu betrachten. PÜ (-)	4	3	2	1	0	
4. Hin und wieder tun mir Menschen sehr leid, wenn sie Probleme haben. EE (-)	4	3	2	1	0	
5. Die Gefühle einer Person in einem Roman kann ich mir oft sehr gut vorstellen. FT	0	1	2	3	4	
6. In Notfallsituationen fühle ich mich ängstlich und unbehaglich. PB	0	1	2	3	4	
7. Ich bleibe gewöhnlich objektiv, wenn ich einen Film oder ein Theaterstück sehe und lasse mich meistens nicht vollkommen von ihm gefangen nehmen. FT (-)	4	3	2	1	0	
8. Ich versuche bei einem Streit zuerst beide Seiten zu verstehen bevor ich eine Entscheidung treffe. PÜ	0	1	2	3	4	
9. Wenn ich sehe, wie jemand ausgenutzt wird, glaube ich ihn schützen zu müssen. EE	0	1	2	3	4	
10. Ich fühle mich hilflos, wenn ich in mitten einer sehr emotionsgeladenen Situationen bin. PB	0	1	2	3	4	
11. Manchmal versuche ich meine Freunde besser zu verstehen, in dem ich mir vorstelle wie die Dinge aus ihrer Perspektive aussehen. PÜ	0	1	2	3	4	
12. Ich bin nur selten von einem guten Buch oder Film sehr gefesselt. FT (-)	4	3	2	1	0	
13. Wenn ich sehe, wie jemand verletzt wird, bleibe ich in der Regel ruhig. PB (-)	4	3	2	1	0	
14. Gewöhnlich geht mir das Unglück anderer Menschen nicht sehr nahe. EE (-)	4	3	2	1	0	
15. Wenn ich sicher bin, recht zu haben, verschwende ich meine Zeit nicht damit mir die Argumente anderer Leute anzuhören. PÜ (-)	4	3	2	1	0	
16. Nach dem ich einen Film gesehen habe, fühle ich mich so, als ob ich eine der Personen aus diesem Film sei. FT	0	1	2	3	4	
17. In einer gespannten emotionalen Situation zu sein, beängstigt mich. PB	0	1	2	3	4	

Universitätsklinikum Essen	Empathietest Auswertung	Institut für Pharmakogenetik

Lfd. Nr.: EMP - 0011 /w/48	nie	selten	manchmal	oft	immer	Ergebnis
18. Wenn ich sehe, dass jemand ungerecht behandelt wird, habe ich manchmal nicht viel Mitleid mit ihm. EE (-)	4	3	2	1	0	
19. Gewöhnlich verhalte ich mich ziemlich umsichtig in Notsituationen. PB (-)	4	3	2	1	0	
20. Mich berühren Dinge sehr, die ich nur beobachte. EE	0	1	2	3	4	
21. Ich glaube jedes Problem hat zwei Seiten und versuche deshalb beide zu berücksichtigen. PÜ	0	1	2	3	4	
22. Ich würde mich selbst als eine ziemlich weichherzige Person bezeichnen. EE	0	1	2	3	4	
23. Wenn ich einen guten Film sehe, kann ich mich sehr leicht in die Hauptperson hineinversetzen. FT	0	1	2	3	4	
24. In heiklen Situationen neige ich dazu die Kontrolle über mich zu verlieren. PB	0	1	2	3	4	
25. Wenn mir das Verhalten eines anderen komisch vorkommt, versuche ich mich für eine Weile in seine Lage zu versetzen. PÜ	0	1	2	3	4	
26. Wenn ich eine interessante Geschichte oder ein gutes Buch lese, versuche ich mir vorzustellen, wie ich mich fühlen würde, wenn mir die Ereignisse passieren würden. FT	0	1	2	3	4	
27. Wenn ich sehe, dass jemand in einem Notfall dringend Hilfe braucht, macht mich das völlig fertig. PB	0	1	2	3	4	
28. Bevor ich jemanden kritisiere, versuche ich mir vorzustellen, wie ich mich an seiner Stelle fühlen würde. PÜ	0	1	2	3	4	

PÜ	0
FT	0
EE	0
PB	0
